# Benevolent and Hostile Sexism in Social Spheres: The Impact of Parents, School and Romance on Belgian Adolescents' Sexist Attitudes

**DOI:** 10.3389/fsoc.2019.00047

**Published:** 2019-05-31

**Authors:** Laora Mastari, Bram Spruyt, Jessy Siongers

**Affiliations:** ^1^Tempus Omnia Revelat (TOR) Research Group, Department of Sociology, Vrije Universiteit Brussel, Brussels, Belgium; ^2^Cultural Diversity: Opportunities and Socialization (CuDOS) Research Group, Department of Sociology, Universiteit Gent, Ghent, Belgium

**Keywords:** benevolent sexism, hostile sexism, sexist attitudes, adolescents, intergenerational transmission, social differences, sociological perspective, gender stereotypes

## Abstract

Despite growing public awareness and policy efforts, gender equality has not yet been fully established in Western societies. Previous research has shown that hostile and benevolent sexist attitudes, which are grounded in traditional gender stereotypes, play a key role in the reproduction of gender inequalities. Whereas, hostile and benevolent sexism among adolescents has been previously studied, limited attention has been paid to social characteristics in understanding the support for these attitudes. In this article, we aim to study how the family, the school and romantic partnerships relate to adolescents' benevolent and hostile sexist attitudes. We relied on data gathered in 2013 by the Flemish Youth Research Platform and performed multivariate analyses on 755 parent-child dyads (*n*_**♂**_ = 342; *n*_♀_ = 413). Our results indicate that social characteristics especially matter to explain the variation in benevolent sexist attitudes among girls and hostile sexist attitudes among boys. Among girls, being in a romantic relationship and parents' traditional moral beliefs was strongly related to benevolent sexism; while for boys, hostile sexism was strongly related to being enrolled in technical and vocational education. In the conclusion, we elaborate on the implications of our findings.

## Introduction

In recent years gender equality has not only received growing public attention, but has also become an important policy topic in Western societies. However, despite enduring efforts, gender equality has not yet been fully established in terms of employment rate, labor market position, payment, participation in decision-making positions, workshare in the household and childcare (The World Bank, 2011; European Union, 2017). Although several causes lay behind these persistent gender inequalities, it is undeniable that traditional gender beliefs and stereotypes (i.e., gender prejudice) play a key role. Very early in life, boys, and girls are taught how to behave, what activities to like or do and what toys or clothing to prefer (Eccles et al., 2000). From the age of 4 to 5 years old, children reveal gender stereotypical preferences with, for example, girls preferring romantic stories and boys leaning toward the more adventurous ones (Durkin and Nugent, 1998). These gender stereotypes carry cultural meanings, practices and (role) expectations that organize life by often (subtly) influencing and guiding people's beliefs, feelings, attitudes, and behaviors (Eckes and Trautner, 2000; Ridgeway and Correll, 2004). During childhood, these gender stereotypes result in boys and girls mainly spending time with same-sex peers and playfully avoiding each other (Powlishta, 2003; Martin and Ruble, 2004). During adolescence this avoidance game disappears due to the emergence of sexual attraction and an interest in intimacy (Maccoby, 1998; Rudman and Glick, 2008). These shifting intergroup relationships render the study of adolescents' sexist attitudes very interesting. Sexist attitudes and beliefs confine and influence future life trajectories by (often subtly) influencing beliefs, feelings, and behaviors (Eckes and Trautner, 2000; Ridgeway and Correll, 2004). They prescribe gender-specific behaviors and roles that hinder young people's ability to discern the variety of emotional, social, and educational capacities and options that can be envisioned (Rainey and Rust, 1999; Paul Halpern and Perry-Jenkins, 2016). Eventually, this undermines (policy) efforts that strive for gender equality (Glick et al., 2001).

In what social contexts do sexist attitudes occur then? Research has not yet thoroughly studied the possible social variation of sexist attitudes among adolescents. Despite having an enormous added value in gaining insight on sexist attitudes, most research on this topic has been primarily carried out by psychologists who often work with relatively small and homogeneous samples. Statistically it has thus been difficult to study the social variation of sexist attitudes. Previous research among (young) adults has already revealed that socio-economic factors such as income, job status and educational attainment affect people's socio-political attitudes (Crompton and Lyonette, 2005; Davis and Greenstein, 2009; Marks et al., 2009). Based on this, we expect that people's social background matters when studying sexist attitudes. Therefore, this study aims to explore how socio-economic and cultural aspects relate to both benevolent and hostile sexist attitudes among adolescents from a sociological perspective. We do this by following Glick and Fiske's (1996) distinction between hostile and benevolent sexist attitudes. To grasp the socio-economic and cultural background of adolescents we differentiate between ascribed and achieved social characteristics.

This paper uses cross-sectional data of 755 parent-child dyads gathered in Flanders (the Dutch-speaking part of Belgium) in 2013 by the Flemish Youth Research Platform. The adolescents were aged between 12 and 18 years old. Research already showed important gender differences in the support for both variants of sexism (Glick et al., 2001; Vandenbossche et al., 2017). Therefore, we studied boys' (*N* = 342) and girls' (*N* = 413) benevolent and hostile sexist attitudes separately.

## Ambivalent Sexist Attitudes

Gender attitudes, such as sexist attitudes, are often based on stereotypical beliefs about gender and can be perceived as a form of prejudice (Rudman and Glick, 2008). In societies that present themselves as tolerant, sexist attitudes take an ambivalent form (Glick and Fiske, 1996, 2011a). Besides the socially less accepted hostile way of expressing sexist attitudes, a benevolent variant has emerged. Therefore, Glick and Fiske (1996) emphasize the ambivalence of sexism, because hostile and benevolent sexism coexist and are theoretically complementary and mutually reinforcing ideologies (Hammond et al., 2017; Cross and Overall, 2018). Both forms of sexism aim the subordination of women, although this is expressed in a different way (Glick and Hilt, 2001).

Hostile sexism aims to preserve men's dominance over women by underlining men's power. It is expressed in a blatant and resentful way toward women who violate traditional roles. Women who don't comply with these traditional (gender) roles are perceived as a threat to men's dominant position. Hostile sexism overtly keeps women in a subordinate position and is even a precursor for sexual harassment and violence toward women (Begany and Milburn, 2002).

Benevolent sexism is a subtler form of sexism and is expressed in a seemingly positive way. It is expressed by emphasizing men's role to protect and provide for women by putting them on a pedestal in a chivalrous way. This protection and love is granted in exchange for women's compliance to traditional gender roles. This form of sexism is instigated through paternal and traditional beliefs that perceive women as beautiful and pure, yet delicate and precious, and therefore in need of protection provided by men (Connelly and Heesacker, 2012; Hayes and Swim, 2013; Cuddy et al., 2015; Vandenbossche et al., 2017; Cross and Overall, 2018). It is this seemingly positive character and the insistence on the complementarity of men and women that makes benevolent sexism a socially more accepted form of sexism. Consequently, it is also an inconspicuous mechanism that perpetuates gender inequality (Glick and Fiske, 2001b; Connelly and Heesacker, 2012). It has been shown that benevolent sexism encourages women to prioritize relationships (family, children, etc.) over pursuing educational or professional goals (see Chen et al., 2009; Montañés et al., 2013) and undermining women's perceptions of their competences and performances (see Dardenne et al., 2007).

Because benevolent sexism is a socially more accepted form of sexism compared to hostile sexism, it is endorsed by both genders. It has been repeatedly demonstrated that both men and women score above average on the benevolent sexism scale, across several countries (de Lemus et al., 2010 and see Glick and Fiske, 2001b cross-country scores on BS and HS). Men and boys consistently score higher on hostile sexism than women and girls (Glick et al., 2001; Becker, 2010; Becker and Wright, 2011; Vandenbossche et al., 2017). More generally, a broad literature has demonstrated that the combination of benevolent and hostile sexism is related to system justification ideologies (Jost and Kay, 2005), which serve as hierarchy-enhancing, legitimizing myths that strengthen group-based inequality (Sibley et al., 2007). This shows that sexism does not occur in a social vacuum where status and power are not relevant, and that studying sexist attitudes from a sociological framework is meaningful.

## Studying Sexist Attitudes From a Sociological Perspective

To study the distribution of sexist attitudes from a sociological perspective, we differentiate between characteristics from the parents (*ascribed social characteristics*) and young people's own social achievements (*achieved social characteristics*) to study young people's benevolent and hostile sexist attitudes.

### Ascribed Social Characteristics: The Parents

Previous research shows that children demonstrate gender stereotypical behavior and preferences from the age of 4 to 5 (see Durkin and Nugent, 1998; Halim et al., 2013; Coyne et al., 2016). This suggests that parents are one of the earliest and vital socialisers of gender conceptions. In the literature, two major interpretations of this socialization process exist. The first considers intergenerational transmission as a process of direct socialization. The *modeling* theory of Bandura (1977), for example, holds that parents function as a role model for children. Similarities between both parties results from observational learning and the modeling of parental behavior and copying attitudes (Bandura, 1977).

The second interpretation of socialization puts forth more indirect ways of transmission. Indirect socialization follows from the shared social conditions of parent and child which influence their beliefs, attitudes and behaviors (Vollebergh et al., 2001; Bengtson et al., 2002; Roest et al., 2010). The direct and indirect pathways of socialization do not rule each other out (Mustillo et al., 2004). In this article, we consider them equally important and study both methods of intergenerational transmission as an overarching concept of the general social climate in which adolescents grow up and sexist attitudes can endure.

#### (More) Direct Ways of Socialization: Parents' Moral and Traditional Gender Beliefs

Direct socialization happens through (verbal) interaction and modeling parents' behavior by demonstrating to children what it means to be male or female (Cunningham, 2001; Davis and Greenstein, 2009). For example, some parents discourage children playing with “sex-inappropriate” toys (Kollmayer et al., 2018) and interact differently with their children by being somewhat rougher with their sons and gentler with their daughters (Eckes and Trautner, 2000; Rudman and Glick, 2008). We assess the more direct ways of parents' socializing influences through their traditional and moral gender beliefs. We consider these beliefs as imbued with stereotypical and traditional values which form a generative climate for children's sexist attitudes. Inglehart's continuum (1997, 2000) shows how moral beliefs are related to traditional beliefs. His continuum consists of a traditional side compared with a secular-rational values side. Individuals who deem the preservation of the family in its traditional structure as important, put a high esteem on traditional gender roles (e.g., women shouldn't make more money than their husbands) and beliefs (e.g., respect toward parents is unconditional) are all placed on the traditional side of Inglehart's continuum. This is alongside individuals who claim that abortion and divorce are unjustifiable. In this article, we conceptualize the latter two as a parent's moral belief, including deeming homosexuality and extramarital sex as unjustifiable. Considering these four topics as unjustifiable stems from the idea that they form a threat to the traditional family structure consisting of a man, woman and a couple of children.

Next to moral beliefs, parents' traditional gender role beliefs are also crucial to take into account because they stereotypically characterize men as task-oriented, assertive and ambitious, while women are associated with affection-oriented characteristics such as kindness, compassion and nurturance (Lin and Billingham, 2014). Gender role expectations in line with these (stereotypical) characteristics can be perceived as traditional and even sexist when differences in gender roles are strongly emphasized. Typically, the main role of women is seen as taking care of the household and children, while men provide the finances. Parents with traditional gender role expectations will believe that certain activities are more appropriate for one gender than the other and will be less likely to encourage their sons and daughters to participate in the same activities (Dumais, 2002). These specific gender role beliefs are likely to foster sexist attitudes among children. We thus expect parents' traditional beliefs to be positively related to children's sexist attitudes. More specifically, we expect that having parents that hold more traditional moral and gender role beliefs, relates to supporting benevolent and hostile sexist attitudes to a greater extent than having more progressive oriented parents.

#### Indirect Ways of Socialization: Parents' Socio-Economic and Cultural Position

Research shows that sexist attitudes are related to socio-economic factors. A lower income (Marks et al., 2009) and manual labor (Crompton and Lyonette, 2005) are associated with more traditional gender expectations. As parent's socio-economic and cultural background determines the environment in which children grow up in, we expect that children's gender beliefs are directly influenced (i.e., irrespective of parents' attitudes) by the characteristics of the social position of their parents (Hello et al., 2004). Based on previous research (Crompton and Lyonette, 2005; Marks et al., 2009), we argue that a lower socio-economic position of the parents (job status, income, etc.) and life conditions that are related to this position, relate to a stronger adherence to traditional gender beliefs and create a climate in which children's sexist attitudes are (socially) embedded.

Beside the purely socio-economical position of the parents, their cultural position (capital) and in particular their educational attainment strongly and negatively relates to traditional gender attitudes (Davis and Greenstein, 2009). We distinguish the cultural position from the socio-economic position, since authors like Houtman (2000) claim that material (socio-economic) and cultural positions should not be combined into a larger concept of “social position” as both may have different effects. It can be understood in the broader framework of the emergence of a society where people's beliefs and behaviors are no longer strongly determined by their economic or material conditions, but where cultural factors become increasingly important predictors of behavior (Elchardus, 2009; de Lange et al., 2015).

In sum, we expect to find a negative relationship between the socio-economic and cultural position of the parents and children's benevolent and hostile sexist attitudes. Additionally, we expect parents' cultural status to be more strongly related to children's benevolent and hostile sexist attitudes than the parents' socio-economic status.

### Achieved Status: The Educational Track

Young people also spend a great share of their time in school which forms an important socialization context. School functions not only as a socializing agent, but also as an institution that puts effort in enhancing (gender) equality, exposes pupils to egalitarian ideas and potentially counters gender stereotypes (Davis and Greenstein, 2009). Moreover, young people's educational trajectory, including one's track position, can be seen as the first and vital step in a young person's own status building. In Flanders (the Dutch-speaking part of Belgium) secondary education consists of four educational tracks: general, arts, technical and vocational education. The general track is considered the most demanding and prestigious track as opposed to the technical track, but especially with regards to the vocational track. The latter two are negatively stereotyped in Western societies (Stevens and Vermeersch, 2010; Boone and Van Houtte, 2013). From 14 years and onwards, which is when pupils have to choose one of these four educational tracks, their future educational career is already strongly predisposed. While academic bachelors are mostly pursued by pupils from the general track, professional bachelors are most often pursued by pupils from technical tracks. Only a small number of pupils from vocational tracks continue their educational career after secondary education (Declercq and Verboven, 2018). Research has also shown that pupils enrolled in technical and vocational education more often report feeling that others look down on them; a feeling which causes a higher sense of futility (Spruyt et al., 2015). Coping with this feeling of being stigmatized has been shown to be related to differences in tastes, behaviors, and attitudes between pupils in different tracks (Elchardus et al., 2013; Van Houtte, 2017; Van Houtte and Boone, 2017). Moreover, a strong gender segregation occurs in the vocational track with specializations such as social care and healthcare for girls, and transport and technology for boys (Lappalainen et al., 2013). With regards to gender beliefs and attitudes, research has repeatedly shown strong differences between the educational tracks with more traditional gender beliefs and the stronger occurrence of sexist attitudes among pupils in the vocational track compared to the general track (Elchardus, 1999; Fernández et al., 2006; de Valk, 2008; Elchardus et al., 2013; Vandenbossche et al., 2017). These stronger traditional gender beliefs are likely to be enforced later on, when they start working in separate spheres with men stereotypically working as, for example, construction workers and women as hairdressers or beauty specialists.

In sum, we expect greater support for benevolent and hostile sexist attitudes among adolescents enrolled in vocational and technical tracks compared to adolescents enrolled in general and arts tracks.

### Intimate Gender Relations Despite Prejudice

We also aim to consider how romance and relationships relate to adolescents' sexist attitudes. Prejudice comes in many forms, but the peculiarity of gender prejudice is the structure of the intergroup relation. The dominant (men) and subordinate (women) groups are founded on interdependence for heterosexual reproduction and romance (Rudman and Glick, 2008). Moreover, gender prejudice intersects with social, ethnic, and religious prejudice showing the persistence and importance of gender prejudice.

The concept of benevolent sexism emerged from the idea that the benevolent form is needed to justify the negativity expressed through hostile sexism, but also as a result of a general disapproval of overtly expressing hostile sexist attitudes (Glick et al., 2001; Viki et al., 2003; Hammond et al., 2017; Cross and Overall, 2018). During childhood, this interdependence is not yet essential for intergroup relations. In contrast, adolescence is accompanied by a rise of sexual attraction and romantic interest in potential partners (Rudman and Glick, 2008). Gender role expectations in close (heterosexual) romantic relationships and dating tend to be distinctly traditional and stereotyped (Viki et al., 2003). Courtly and chivalrous (yet sexist) male behavior is highly appreciated by women, while passive and delicate behavior on the female side (acting like a “princess”) is expected by men (Glick and Fiske, 2001b; Serewicz and Gale, 2008; Bohner et al., 2010). Benevolent sexism emphasizes the notion and romantic idea that men and women are two parts of a whole (Glick and Fiske, 2001b). Previous studies have shown that experiences with romantic relationships relates to supporting benevolent sexist attitudes to a higher extent (de Lemus et al., 2010; Lee et al., 2010; Hammond et al., 2016). The dangers of these “romantic,” yet sexist beliefs, are that interdependency and complementarity are promoted. Implicitly, women are continuously being subordinated and gender equality is discouraged by persuading women that protection and love will be granted to them (by men) if they comply with these traditional and sexist beliefs. If not, men will have to react with hostile sexist attitudes in order to preserve the status quo (de Lemus et al., 2010).

In sum, we expect adolescents with a romantic partner to support benevolent sexist attitudes to a higher extent than adolescents without a romantic partner. Based on the literature we do not expect such an association for hostile sexist attitudes. But because benevolent and hostile sexism are related to each other, we study whether being in a romantic relationship relates to a greater support for hostile sexist attitudes.

***Overview of hypotheses***

- Having parents that hold more traditional gender and moral beliefs, relates to a greater support for benevolent and hostile sexism.
- Having parents with a lower socio-economic and especially a lower cultural position, relates to a greater support for benevolent and hostile sexism.
- Being enrolled in vocational and technical tracks relates to a greater support for benevolent and hostile sexism.
- Having a romantic partner relates to a greater support for both forms of sexism, but especially for benevolent sexism.

## Materials and Methods

For our analyses, we relied on data of the “school-monitor” gathered in 2013 by the Flemish Youth Research Platform (JOP: http://www.jeugdonderzoeksplatform.be/en/) among pupils in 98 Flemish secondary schools. All educational tracks and grades were represented in the sample (Bradt et al., 2014). In general, respondents were 12 to 18 years old with an average age of 15.8 (*SD* = 1.62)^1^. The response rates at school level and pupil level were 44.7 and 88%, respectively.

This study did not apply for ethical advice for several reasons. First, our study did not include any medical treatment and Belgium's national regulations, nor the Vrije Universiteit Brussel oblige ethical approval of the research design. Moreover, in 2013 no ethical commission for the humanities existed at the Vrije Universiteit Brussel, making it impossible to ask for ethical advice for the data collection of this study at the Vrije Universiteit Brussel. However, in 2018 a positive ethical advice was granted from SMEC (Sociaal-Maatschappelijke Etnische Committee of the Catholic University of Leuven) to the new edition of this survey. The latter followed exactly the same principles and practices as the data that were gathered in 2013 (the data used in this paper). Before respondents were asked whether they would be willing to fill out the questionnaire, they were explained that participation to the study was voluntary and anonymous. They could stop their participation at any moment without giving any reason and did not have to answer questions that they did not want to answer. Parents who did not want their child to participate in the study, had the opportunity to sign a form stating their child will not take part in the study. These notes were distributed about 2 weeks before the survey took place.

The pupils also received a questionnaire which they could take home for one of their parents to fill out. In our study, only those respondents were selected of whom a parent had also completed a questionnaire and we solely used pupils with a Belgian father, mother and grandmother (Belgian native adolescents). We did this because the non-response analysis showed a certain selectivity with regards to social background (pupils had to fill out the educational level of the parents) and ethnic background. The response was low and therefore very selective among the latter, which is why we chose to focus on parent-child dyads with a Belgian background. After deleting cases who had missing values for one of the variables included in the analyses, we ended up with a final sample of 755 parent-child dyads (adolescents: *n*_**♂**_ = 342 and *n*_**♀**_ = 413; parents: *n*_**♂**_ = 173 and *n*_**♀**_ = 582). Previous research showed benevolent and hostile sexism works in distinct ways for boys and girls (Vandenbossche et al., 2017). Therefore, analyses were performed for boys and girls separately. As the intra-class correlation coefficient only showed weak variation at the level of the schools^2^, we performed unilevel regression analyses.

### Dependent Variables: Benevolent and Hostile Sexism

The dependent variables were benevolent sexism (BS) and hostile sexism (HS) toward women. The items of both scales were derived from the “Ambivalent Sexism Inventory” by Glick and Fiske (1996). Originally the inventory consisted of 22 items measuring hostile and benevolent sexism (each 11 items). Since our data was gathered by means of a (school) survey consisting of varying questions and subjects, we worked with a reduced scale. Theoretically, the benevolent sexism scale consists of three subscales: heterosexual intimacy^a^ (4 items), protective paternalism^b^ (4 items), and complementary gender differentiation^c^ (3 items). We selected items of which the pretest of the questionnaire among a small convenience sample indicated that they had the clearest meaning for young adolescents. We added ‘Compared to men, women are more honest’ to include a stereotypical gender trait. Honesty and sincerity have been linked to typical female stereotypes and sexist beliefs (Dolan, 2014; Etchezahar and Ungaretti, 2014). Respondents rated four items for each form of sexism on a Likert scale ranging from one (totally disagree) to five (totally agree). The internal consistency of the benevolent sexism scale, however, was relatively low (Cronbach α = 0.625). Principal components analysis (PCA) on the four items revealed two components with Eigenvalue above one (1.887 and 1.278). Although the component loadings of a one-dimensional solution were acceptable (loadings > 0.654), subsequent reliability analysis suggested that constructing two correlated scales consisting of two items each, better fitted our data. The first subscale is composed by the items tapping into protective paternalism and heterosexual intimacy. This subscale can be described as benevolent sexism through gender interdependence between (Cronbach α = 0.720). The second component is composed by ‘Compared to men, women are more honest’ and ‘Women have a quality of purity few men possess’. This subscale emphasizes the differences between both genders and can best be described as benevolent sexism through gender essentialism (Cronbach α = 0.750). As a concept, gender essentialism is about the intrinsic qualities that are proposed as natural and fixed. It can be argued that the idea of gender essentialism justifies (romantic) interdependence. Together, the two subscales thus grasp the idea of benevolent sexism well. We performed our analyses on the composed measure for benevolent sexism and for the two separate subscales. Hostile sexism consisted of one factor and was internally consistent (Cronbach α = 0.740; Eigenvalue = 2.248).

### Independent Variables

#### Socio-Economic and Cultural Position

Parents' socio-economic position was operationalized by means of a categorical principal components analysis (CATPCA) and was based on several characteristics: income deprivation according to the child, the employment status of the mother/father according to the child (fulltime, part time, etc.), renting or owning the home, the parent's^3^ experiences with unemployment, the parent's job title (laborer, employee,…) and employment status (Cronbach α = 0.618; Eigenvalue = 2.128). Parents' educational degree was measured by distinguishing between children of whom both parents obtained a master or bachelor degree (n = 479) and children of whom only one parent had a degree of tertiary education (n = 276) with the latter being the reference group.

#### Traditional Gender and Moral Beliefs

Parents' traditional gender role beliefs were measured by the following five items (rated on a 5 point Likert-scale ranging from strongly disagree—strongly agree) (Cronbach α = 0.804): ‘It’s best when a woman takes care of the household and the man is the breadwinner', ‘A woman should stop working and stay at home when she has small children’, ‘You can raise boys more freely than girls’, ‘A woman is better suited to raise small children than a man’, and ‘It's less important for a girl to get a good education than for a boy’. The items originate from the “Onderzoek naar Waardeopvoeding 1996/1997” [Research into value education 1996/1997] (Elchardus, 1999). Item scores were summed and rescaled to a 0–100 continuum.

Parents' moral beliefs were also constructed using a summated scale (0–100) that goes from totally not acceptable to totally acceptable. The four items that were used were adapted from the World Value Survey, rated on a Likert scale (1–5) and referred to acceptance of divorce, extramarital sex, homosexuality, and abortion (Cronbach α = 0.686; see Table 3).

#### Educational Track (and Grade/Age)

The educational track was measured by distinguishing between pupils in general or arts secondary education and pupils in technical or vocational secondary education (0: technical and vocational track, n = 409; 1: general and arts track, n = 346). We took the grade the pupils were enrolled in into account as a continuous, control variable (going from the 1st grade to the 6th grade; M = 4.18, SD = 1.08). We expected pupils' attitudes to be more similar in the same grade rather than in the same year of age, because pupils in the same grade are confronted with similar learning experiences and other events (e.g., senior prom).

#### Romantic Partner

Since the survey did not contain a straightforward question concerning whether the adolescents had a romantic partner or not, we used an alternative question where they were asked to indicate whom they could turn to when needed. One of the options here was the partner/girl- or boyfriend. The option ‘not applicable’ was also available. This made it possible to filter out the adolescents who do not have a romantic partner (0: no partner, n = 304; 1: partner, n = 372).

## Results

Table 1 presents, for boys and girls, the frequencies and mean scores on the separate items of the benevolent and hostile sexism scales. One-way ANOVA tests showed significant differences between girls and boys with regards to hostile sexism F_(1,753)_ = 70.23, p = 0.00) benevolent sexism F_(1,753)_ = 5.51, p = 0.02), benevolent sexism through gender interdependence F_(1,742)_ = 8.45, p = 0.01), but not for benevolent sexism through gender essentialism F_(1,751)_ = 0.63, p = 0.43. Girls scored higher on the items tapping into benevolent sexism than boys, while the opposite was found for hostile sexism where the mean scores on the items were higher for boys. For the first item of benevolent sexism (‘Every man ought to have a woman he adores’), the mean difference between boys and girls was not significant F_(1,752)_ = 1.47, p = 0.23. Table 2 presents the mean scores on the BS and HS scales for boys and girls separately. Girls scored only slightly higher on the composed benevolent sexism scale than boys. Girls also scored higher for benevolent sexism through gender interdependence than boys and for benevolent sexism through gender essentialism than boys. On the other hand, boys clearly scored higher on hostile sexism than girls.

**Table 1:** Frequencies and means for items tapping into benevolent and hostile sexism for boys and girls separately.

		**Frequencies (%)**					
		**(Totally) Disagree**	**Undecided**	**(Totally) Agree**	**Mean (1–5)**	**Standard Deviation**	**Sig. (*****p*****-value) of mean difference between**_**♂**_ **and**_**♀**_
**BENEVOLENT SEXISM TOWARD WOMEN**							
**Gender interdependence**							
Every man ought to have a woman he adores^a^	_**♂**_	17.4	37.6	45.0	3.30	0.96	0.23
	_♀_	24.4	33.1	42.6	3.21	1.02	
A good woman should be set on a pedestal^b^	_**♂**_	11.5	31.8	56.7	3.50	0.88	0.005
	_♀_	9.7	25.1	65.2	3.68	0.88	
**Gender essentialism**							
Compared to men, women are more honest	_**♂**_	46.4	37.5	16.1	2.60	0.92	0.007
	_♀_	38.0	42.6	19.4	2.78	0.93	
Women have a quality of purity few men possess^c^	_**♂**_	43.4	40.7	15.9	2.62	0.90	0.01
	_♀_	37.3	44.1	18.7	2.78	0.88	
**HOSTILE SEXISM TOWARD WOMEN**							
Women seek special favors under guise of equality	_**♂**_	24.5	44.5	30.9	3.08	0.88	< 0.001
	_♀_	48.9	33.4	17.6	2.59	0.92	
Women fail to appreciate all men do for them	_**♂**_	18.2	42.2	39.6	3.26	0.86	< 0.001
	_♀_	41.1	33.6	25.3	2.79	0.98	
Once a man commits, she puts him on a tight leash	_**♂**_	27.3	43.0	29.0	3.02	0.89	< 0.001
	_♀_	45.9	33.8	20.3	2.69	0.92	
Women are too easily offended	_**♂**_	7.9	32.6	59.5	3.63	0.84	< 0.001
	_♀_	18.4	36.3	45.3	3.29	0.91	
**BIVARIATE CORRELATIONS BETWEEN SEXISM SCALES**							
Benevolent sexism and hostile sexism	0.25^**^						
Gender interdependence and hostile sexism	0.09^*^						
Gender essentialism and hostile sexism	0.10^*^						
Gender interdependence and essentialism	0.19^**^						

Significance levels:

** p < 0.010.

* p < 0.050.

*Items are ranging from 1 to 5, for ease of presentation the outer categories were taken together*.

a *Heterosexual intimacy*.

b Protective paternalism.

c *Complementary gender differentiation*.

**Table 2:** Means for subscales and scales of benevolent and hostile sexism for boys and girls separately.

		**Mean (0–100)**	**Standard Deviation**	**Sig. (p-value) of mean difference between _**♂**_and _**♀**_**
Benevolent sexism toward women	_**♂**_	50.20	15.92	0.02
	_♀_	52.93	15.85	
Gender interdependence	_**♂**_	40.16	20.04	0.004
	_♀_	44.51	20.55	
Gender essentialism	_**♂**_	59.99	20.74	
	_♀_	61.20	20.74	0.43
Hostile sexism	_**♂**_	56.16	16.02	
	_♀_	46.01	17.00	< 0.001

**Table 3:** Frequencies and means for items tapping into parent's traditional gender and moral beliefs (N = 755).

		**Frequencies (%)**				
		**(Totally) disagree**	**Un-decided**	**(Totally) agree**	**Mean (1–5)**	**Standard Deviation**
**TRADITIONAL GENDER BELIEFS**						
It's best when a woman takes care of the household and the man is the breadwinner		91.1	6.8	2.2	1.57	0.74
A woman should stop working and stay at home when she has small children		82.2	11.9	5.8	1.81	0.92
You can raise boys more freely than girls		91.9	6.5	1.6	1.61	0.69
A woman is better suited to raise small children than a man		73.1	16.4	10.5	2.05	0.99
It's less important for a girl to get a good education than for a boy		97.6	1.5	1.0	1.36	0.58
Eigenvalue	2.90					
Cronbach α	0.80					
		**(Totally) Unaccept-able**	**Neutral**	**(Totally) Accept-able**	**Mean (1–5)**	**Standard Deviation**
**MORAL BELIEFS TOWARD …**						
Divorce		3.7	19.2	77.1	3.97	0.81
Extramarital sex		68.4	23.8	7.8	2.12	0.94
Homosexuality		3.7	11.0	85.3	4.12	0.82
Abortion		9.9	27.7	62.3	3.61	0.92
Eigenvalue	2.12					
Cronbach α	0.69					

Table 4 presents the results of the stepwise (forced entry) multivariate regression analyses with standardized beta coefficients for benevolent sexism (row a), the benevolent sexism through gender interdependence subscale (row b) and the benevolent sexism through gender essentialism subscale (row c) for boys and girls separately. In the first model, we included the socio-economical position of the parent and parents' educational degree, in the second model we added parent's traditional gender and progressive moral beliefs. Next, we added the adolescents' age (grade) and educational track, and in the final model we introduced whether one had a romantic partner or not^4^.

**Table 4:** Multivariate regression analysis for benevolent sexism and subscales for boys (N = 342) and girls (N = 413).

		**MODEL 1**		**MODEL 2**		**MODEL 3**		**MODEL 4**	
		**Boys**	**Girls**	**Boys**	**Girls**	**Boys**	**Girls**	**Boys**	**Girls**
		**Stand. Beta**	**Stand. Beta**	**Stand. Beta**	**Stand. Beta**	**Stand. Beta**	**Stand. Beta**	**Stand. Beta**	**Stand. Beta**
**Socio-economical status (SES)**	a	−0.04	−0.09(^*^)	−0.04	−0.06	−0.02	−0.05	−0.04	−0.04
	b	−0.00	−0.04	0.01	−0.03	0.02	−0.01	0.02	−0.00
	c	−0.05	0.10(^*^)	−0.06	−0.08	−0.05	−0.07	−0.08	−0.07
**Both parents higher educational**	a	−0.09	−0.13^**^	−0.08	−0.10^*^	−0.06	−0.06	−0.03	0.06
**degree**	b	−0.12^*^	−0.21^***^	−0.11(^*^)	−0.18^***^	−0.08	−0.14^**^	−0.08	−0.14^*^
(0: only one parent higher degree)	c	−0.02	0.01	0.00	0.04	−0.00	0.06	0.02	0.06
**Parent's traditional**	a			0.00	0.10(^*^)	0.01	0.10(^*^)	−0.04	0.09
**gender beliefs**	b			0.02	0.04	0.03	0.04	0.01	0.03
	c			−0.04	0.12^*^	−0.05	0.12^*^	−0.08	0.12^*^
**Parent's progressive moral beliefs**	a			−0.09	−0.14^**^	−0.09	−0.13^*^	−0.11(^*^)	−0.13^*^
	b			−0.08	−0.15^**^	−0.08	−0.13^*^	−0.07	−0.14^*^
	c			−0.15^*^	−0.09(^*^)	−0.15^*^	−0.09	−0.17^**^	−0.07
**Grade** (age)	a					0.03	−0.12^*^	−0.00	−0.12^*^
	b					−0.07	−0.15^***^	−0.09	−0.15^**^
	c					0.10(^*^)	−0.02	0.08	−0.03
**Educational track**	a					0.08	0.09(^*^)	0.10	0.07
(0: general and arts track)	b					0.10(^*^)	0.10(^*^)	0.13^*^	0.07
	c					0.00	0.04	0.00	0.03
**Romantic partner**	a							0.08	0.14^**^
(0: no romantic partner)	b							0.04	0.09(^*^)
	c							0.10(^*^)	0.12^*^
**R**^**2**^ **Adjusted**	a	0.01	0.03	0.01	0.07	0.01	0.08	0.01	0.09
	b	0.01	0.05	0.01	0.07	0.02	0.10	0.02	0.09
	c	0.00	0.01	0.01	0.03	0.02	0.03	0.02	0.03

*Significance levels*:

*** p < 0.001.

** *p < 0.010*.

* p < 0.050

(*) *< 0.100*.

a) Benevolent sexism (gender interdependence and essentialism).

b) Benevolent sexism through gender interdependence.

*c) Benevolent sexism through gender essentialism*.

For boys, the results showed a clear and significant effect for the parent's progressive moral beliefs for the support of benevolent sexism through gender essentialism and benevolent sexism. Furthermore, the results indicated that the support for gender essentialism, was positively related to boys' age. This effect disappeared, however, by adding whether boys had a romantic partner or not in the final model. Although the effect was relatively weak and borderline significant, the results indicated that boys with a romantic partner supported gender essentialism to a higher extent than boys without a romantic partner. The support for gender interdependence showed to be negatively related to parents' educational degree in the first and second model. This effect disappeared, by adding adolescents' educational track in the third model and final model. Boys enrolled in technical and vocational tracks thus supported gender interdependence to a higher degree than boys enrolled in general and arts tracks.

For girls, the first model showed that parents' educational degree was negatively related to benevolent sexism and the gender interdependence subscale. The strength of the effect decreased in model 2 when the parent's progressive moral beliefs were entered. However, in the final model, a clear and significant effect remained for the gender interdependence subscale. Moreover, the results showed that in the final model, parent's progressive moral beliefs related to girls' benevolent sexist attitudes and their attitudes with regards to gender interdependence. Furthermore, the results showed that girls' support for benevolent sexism decreased with age. While in model 3 the results showed a significant effect of the parent's traditional gender beliefs in explaining variation in benevolent sexist attitudes for girls, this was no longer the case in the final model when we controlled for having a romantic partner. However, parents' traditional gender beliefs continued to relate to girls' attitudes with regards to gender essentialism in the final model. Finally, the results showed that for girls; having a romantic partner related to supporting benevolent sexist attitudes, gender interdependence and gender essentialism to a higher extent than boys. For boys, this was only the case for benevolent sexism through gender essentialism. From a more general perspective, it is noteworthy that for benevolent sexism and benevolent sexism through interdependence, the explanatory power of all variables included in the final model, is higher for girls (Adjusted R^2^ = 0.09, F_(7,375)_ = 6.39, p < 0.001 and Adjusted R^2^ = 0.10, F_(7,372)_ = 6.69, p < 0.001 respectively) than for boys (Adjusted R^2^ = 0.01, F_(7,284)_ = 1.86, p < 0.05 and Adjusted R^2^ = 0.02, F_(7,285)_ = 1.94, p < 0.05).

Table 5 presents the results for the same analyses for hostile sexism. The results were unclear for girls, except in the first and second model where parents' educational degree was negatively related to hostile sexist attitudes. However, this effect fades in the third and final model. No significant effects were found in the final model for girls. For boys (similarly to girls), the first model demonstrated that parents' educational degree rather than the parent's socio-economical background related to hostile sexist attitudes. By adding the parent's traditional gender and progressive moral beliefs in the second model (for boys), the effect of parents' educational degree seemed to fade. This indicates that the effect of the educational degree of the parents was channeled by their moral beliefs. Furthermore, model 3 and especially the final model clearly showed that for boys, the educational track one was enrolled in explained a substantial share of the variance in hostile sexist attitudes. The results indicated that the occurrence of hostile sexist attitudes was higher among boys enrolled in technical and vocational tracks compared to boys enrolled in the general and arts tracks. The predictive power of the final model was higher among boys (*Adjusted R*^2^ = 0.06, *F*_(7,285)_ = 3.72, *p* < 0.001) when compared to girls (*Adjusted R*^2^ = 0.03, *F*_(7,375)_ = 2.74, *p* < 0.01). The gender difference on this point was more modest when compared to benevolent sexism, but the pattern is completely opposite.

**Table 5:** Multivariate regression analysis for hostile sexism for boys (*N* = 342) and girls (*N* = 413).

	**MODEL 1**		**MODEL 2**		**MODEL 3**		**MODEL 4**	
	**Boys**	**Girls**	**Boys**	**Girls**	**Boys**	**Girls**	**Boys**	**Girls**
	**Stand. Beta**	**Stand. Beta**	**Stand. Beta**	**Stand. Beta**	**Stand. Beta**	**Stand. Beta**	**Stand. Beta**	**Stand. Beta**
Socio-economical status (SES)	0.02	−0.10(^*^)	0.03	−0.08	0.06	−0.07	0.90	−0.05
Both parents higher educational degree (0: only one parent higher degree)	−0.10(^*^)	−0.12^*^	−0.09	−0.10^*^	−0.05	−0.07	−0.04	−0.08
Parent's traditional gender beliefs			0.03	0.07	0.04	0.07	0.06	0.06
Parent's progressive moral beliefs			−0.12^*^	−0.06	−0.12^*^	−0.05	−0.14^*^	−0.06
Grade (age)					0.05	−0.02	0.06	−0.07
Educational track (0: general and arts tracks)					0.18^**^	0.09	0.20^***^	0.09
Romantic partner (0: no romantic partner)							0.03	0.04
R^2^ Adjusted	0.00	0.03	0.02	0.03	0.04	0.04	0.06	0.03

*Significance levels*:

*** *p < 0.001*,

** *p < 0.010*,

* p < 0.050

(*) *< 0.100*.

In general, the results suggest that benevolent sexist attitudes can be explained through their social characteristics for girls but to a much lesser extent for boys. On the contrary, hostile sexist attitudes can be explained through social characteristics for boys, but not so much for girls.

## Discussion

In this paper we studied (social) differences in the support for hostile and benevolent sexism based on 755 parent child-parent dyads. Based on the different items for benevolent sexism, two subscales were defined, i.e., benevolent sexism through gender interdependence and gender essentialism. In this section, we discuss the implications of our results and suggest directions for further research.

### Implications of the Results

Our data did not allow to make any statements with regards to the causality of the associations, therefore we focused on how they relate to each other. First, our results showed that the educational degrees of the parents (cultural status) relate more strongly to sexist attitudes than their socio-economic status. This is in line with the idea that cultural factors have become increasingly important predictors of attitudes in contemporary societies (Elchardus, 2009; de Lange et al., 2015). This is also a first indication that sexist attitudes are better interpreted from a socio-cultural framework than from a purely socio-economic one. Continuing on the relevance of parents to understand children's sexist attitudes, our results revealed that for girls, parent's traditional gender beliefs are related to girls' support for gender essentialism. Parent's traditional gender beliefs are constituted of gender role beliefs that are based on this gender essentialism. It is possible that this emphasis on gender roles affects girls more, because it is more directed to them. Overall, but with exception to gender interdependence for boys and gender essentialism for girls, parent's moral beliefs relate more strongly to children's benevolent and hostile sexist attitudes, than to their traditional gender beliefs. Following Inglehart (1997, 2000), moral beliefs are strongly endowed with cultural and traditional meanings and values with regards to close relationships, while as stated earlier, traditional gender beliefs are more concerned with role expectations toward men and women. Moral beliefs are constituted by powerful beliefs with regards to normative values and rules and contain less ambiguity when compared to beliefs about traditional gender roles. It is possible that this explains why traditional gender beliefs of parents are less transmitted to adolescents, while moral beliefs about topics like divorce, extramarital sex, homosexuality and abortion are more tangible (Meeusen and Boonen, 2017).

Secondly, while the ascribed social position of adolescents does not seem to relate directly to their sexist attitudes (although the parent's attitudes are strongly related to their social position), their own provisionally achieved position (i.e., their educational track position) does predict sexist attitudes and more precisely boys' hostile sexist attitudes and the support for gender interdependence. Previous research showed differences in sexist attitudes among adolescents of different educational tracks (see Vandenbossche et al., 2017). This association can be interpreted as a self-reinforcing mechanism. Adolescents following school in vocational and technical tracks often share a similar ascribed social background with regards to parents' educational degrees. Both among parents and children lower educational degrees have been associated with more traditional gender beliefs. It can be argued that adolescents following school in vocational and technical tracks a priori support sexist attitudes to a higher extent compared to adolescents in general and arts tracks. Being around other adolescents with similar backgrounds and beliefs, may reinforce these beliefs. Moreover, vocational and technical tracks in Flanders are characterized by a strong gender segregation with regards to the gender specific offered courses (Van Houtte, 2004). However, this cannot solely be explained by these aspects. Against the background of knowledge societies, the social position and stigma accompanied with the technical and especially vocational tracks in Flanders generate feelings of being looked down on (see Spruyt et al., 2015). It is possible that they try to compensate for their less advantageous social position which may enhance their motivation to gain social status through gender relations. Consequently, it is possible that these pupils react to this stigma by adopting hostile sexist attitudes since these are among the attitudes that contrast those promoted by schools. Furthermore, boys experience negative stereotypes and felt stigma more often than girls (Spruyt et al., 2015).

Finally, our results show that especially for girls, being in a romantic relationship strongly relates to supporting benevolent sexist attitudes to a higher extent compared to girls who are not in a romantic relationship. This suggests that for girls, benevolent sexism truly is about the romantic and chivalrous part (the whole concept of a ‘Prince Charming’). Since we worked with cross-sectional data, we were unable to test whether this is due to socialization or selection effects, although it is very clear that being in a relationship does strongly relate to girls' benevolent sexist attitudes. On the one hand, if socialization effects are at play, this would imply that girls' benevolent sexist attitudes are strengthened when being in a romantic relationship. Hammond et al. (2016) longitudinal research indeed showed that women's benevolent sexist attitudes changed proportionally over the course of the relationship with their partners' benevolent sexist attitudes. Of all interrelations, romantic relationships are the most ideal circumstances in which benevolent sexism can prevail and thus not be seen as sexism (Barreto and Ellemers, 2005). Moreover, with regards to romance and relationships, cultural ideals about how men and women ‘should’ interact with each other are widespread (Viki et al., 2003; Serewicz and Gale, 2008).

### Limitations and Further Directions

One of the important limitations of this study is that when studying the role of the parents in the transmission of attitudes, the gender of the parent may matter. However, the data we worked with did not allow us to report reliable results due to the relatively small (sub-)samples and skewed distribution of the gender of the parent that filled out the questionnaire (predominantly mothers). In the literature, different hypotheses exist with regards to the gender of the parent and the influence on their children's attitudes, which is also commonly referred to as parent-child similarity (Meeusen and Boonen, 2017). One of these is the ‘mother’s dominance' hypothesis which states that child-mother similarity with respect to attitudes will be more pronounced than child-father similarity. Mothers are thought to have more influence in the formation of children's attitudes because they are more involved with their upbringing (Jaspers et al., 2008; Degner and Dalege, 2013). Another hypothesis is the gender-matching hypothesis which expects daughters to resemble their mothers more than their father and sons to resemble their fathers more than their mothers (Nieuwbeerta and Wittebrood, 1995). Thus, future research on the intergenerational transmission of gender (and sexist) attitudes should take both parents into account in order to compare effects of parent's gender. We were unable to perform such analyses due to a lack of information about both parents.

A second limitation related to our data is the indirect way through which we operationalized being in a romantic relationship. The questionnaire did not present a direct question with regards to the relational status of the adolescents. Therefore, in our analyses we could only distinguish adolescents that considered their partner as someone they could turn to when needed and those who did not. It is possible that our measure of being in a romantic relationship is somewhat biased toward adolescents who had a more “serious” relationship. Research should perceive romantic relationships (and as such the entire dating world) as a crucial study context where sexism can carelessly manifest itself. Further research should consider different kinds of relationships where more or less traditional gender concepts are at play (see Lee et al., 2010; Hammond and Overall, 2015), but also how this relates to other aspects like social and cultural position. Couples should be studied in a longitudinal way by taking into account how sexist attitudes change over the course of a relationship and how similar couples are (or become) in their attitudes. de Lemus et al. (2010) study showed that adolescents with more romantic relationship experience tended to endorse higher degrees of sexism. They found higher degrees of hostile sexism among boys. This article adds to the literature by showing that for girls, benevolent sexism relates to being in a romantic relationship. This may also indicate that the development of sexist attitudes peaks when experiencing a romantic relationship, although differently for boys and girls. On the other hand, it is possible that selection effects are at play. Adolescent girls who have been socialized into benevolent sexist attitudes may more easily opt to be in a relationship, longing for their ‘Prince Charming’ and may at the same time be more appealing to boys (de Lemus et al., 2010). In turn, Bohner et al. (2010) showed that likability ratings among women are highest toward men who showed to endorse benevolent sexist attitudes. It seems as if benevolent sexism, or at least its chivalrous side, is attractive to young adolescent girls. The downside of benevolent sexism, however, is that it is founded on traditional and unequal gender stereotypes that implicitly block gender equality. Women should be taught to challenge these stereotypes and to perceive romance as something that should not get in the way of pursuing other (educational, career, etc.) goals (see Hammond and Overall, 2015).

## Conclusion

This article studied adolescents' sexist attitudes from a sociological perspective. The results showed that differences occur in the endorsement of sexist attitudes with regards to adolescent boys' and girls' social characteristics. In sum, it seems that support for benevolent sexist attitudes was less likely for girls who weren't romantically involved than for girls who were. Girls also supported benevolent sexist attitudes less the older they got. Parents' moral beliefs related to benevolent sexist attitudes for both boys and girls. Having a parent who thinks divorce, extramarital sex, homosexuality, and abortion are justifiable creates a climate where benevolent sexist attitudes are endorsed to a lesser extent. This was also the case for boys, with regards to hostile sexist attitudes. The educational track adolescents are enrolled in, related to boys' hostile and benevolent sexist attitudes (gender interdependence). With regards to girls' hostile sexist attitudes, no clear results were found. Based upon the results of this study, we can conclude that social characteristics especially matter to explain the variation in benevolent sexist attitudes among girls and hostile sexist attitudes among boys.

## Author Contributions

LM, BS, and JS wrote this article, performed statistical analysis and discussed the results together.

### Conflict of Interest Statement

The authors declare that the research was conducted in the absence of any commercial or financial relationships that could be construed as a potential conflict of interest.

## Footnotes

^1^With some outliers up to 23-year olds that were not withheld for analyses.

^2^For boys, the intraclass correlation coefficient (ICC) for benevolent sexism was 6.02%, for benevolent sexism through gender interdependence the ICC was 6.29% and for benevolent sexism through gender essentialism the ICC 7.19%. For girls the ICC was lower than 1% for composed benevolent sexism and subscales. For boys, the ICC for hostile sexism was lower than 1% and for girls the ICC = 6.22%. Robustness checks with multilevel models are available upon request.

^3^This refers to the parent who filled out the questionnaire. We only possess information about both parents with regards to the educational degree.

^4^To exclude multicollinearity, we checked the variance inflation factors (VIF). The VIF's indicate the absence of multicollinearity (the VIF's vary between 1.016 and 1.257 for the final models).
